# Prostatic Abscesses in a Patient Receiving Tumor Necrosis Factor-Alpha Inhibitor Therapy for Hidradenitis Suppurativa: A Case Report

**DOI:** 10.7759/cureus.41820

**Published:** 2023-07-13

**Authors:** Severin Lautenschlager, Räto Strebel, Khosrow Ahmadi, Jan Birzele, Alexander Gu, Anna Nowag, Thomas Scherer, Uwe Bieri

**Affiliations:** 1 Department of Urology, Cantonal Hospital of Graubünden, Chur, CHE; 2 Department of Urology, University Hospital Zurich, Zurich, CHE

**Keywords:** adverse drug events, prostatitis, tnf-alpha blocker, immunomodulating agents, abscess

## Abstract

This report is the first to present the case of a patient who developed bacterial abscess-forming prostatitis while undergoing treatment with adalimumab, a tumor necrosis factor-alpha blocking therapy, for hidradenitis suppurativa. A 36-year-old male presented with persistent anogenital pain and dysuria for approximately three weeks. Two days before presentation at the emergency room (ER), a rubber band ligation was performed to address suspected hemorrhoids stages I-II. In the ER, clinical and laboratory examinations suggested acute prostatitis, prompting the initiation of antibiotic therapy. In the absence of an adequate response, magnetic resonance imaging was performed, which identified a complex abscess and fistulation system originating from the right prostatic lobe. Following the insertion of a drain, adalimumab was discontinued, and antibiotic therapy was intensified, resulting in the resolution of the abscess. After six weeks, follow-up showed the patient to be free of symptoms. This case highlights a rare adverse event of patients using immunomodulating medications and may help physicians to manage similar cases in the future. Immunomodulating drugs can lead to the development of prostatic abscesses in young patients, necessitating attentive and careful clinical examination with a low threshold for further diagnostic workup in uncommon case presentations.

## Introduction

Adalimumab is a fully human monoclonal antibody targeting tumor necrosis factor-alpha (TNF-α) and is a widely used anti-inflammatory biological drug that is well-established for the treatment of hidradenitis suppurativa [1,2]. Also known as acne inversa, hidradenitis suppurativa is a painful, chronic inflammatory skin disease, characterized by deep-seated lesions in the apocrine gland-bearing areas of the body, predominantly affecting the axillary, inguinal, and anogenital regions [3]. Due to immunomodulating effects, TNF-α blockade may increase the incidence of adverse events, such as respiratory, urinary, or skin infections [4]. A prostatic abscess is a rare but serious condition, resulting from the accumulation of pus within the prostate gland and is commonly caused by *Escherichia coli*, *Klebsiella pneumoniae*, or *Staphylococcus aureus* [5]. To our knowledge, this report presents an unprecedented case of an individual developing acute prostatitis with abscess during anti-TNF-α therapy with adalimumab.

## Case presentation

A 36-year-old male Caucasian presented at the emergency room (ER) due to anogenital pain, radiating to the scrotal region and burning during urination. The patient had reported similar complaints two weeks earlier, which were treated by the family doctor with antibiotics who suspected a urinary tract infection. Because of persistent symptoms, the patient was assigned to a gastroenterologist, who performed a rubber band ligation to treat hemorrhoids stage I-II two days before presentation at the emergency ward. The patient’s medical history revealed hidradenitis suppurativa treated with adalimumab for about five years, arrhythmogenic heart disease with an implanted pacemaker, and epilepsy treated with levetiracetam. The patient has no history of prior urogenital interventions. Clinically, a firm elastic prostate with tenderness on palpation was present. Fever or suprapubic pressure pain was absent. Laboratory workup revealed elevated inflammatory markers, with a C-reactive protein of 31 mg/L (<5 mg/L) and a white blood cell count of 14.1 × 10^3^/µL (3.9-10.2 × 10^3^/µL). Renal function was not impaired. Urinanalysis was remarkable for leukocyturia and bacteriuria, and mid-stream urine was cultured. Screening for sexually transmitted diseases was negative, and an abdominal sonographic examination of the urinary tract and testis showed normal findings. Due to a suspected diagnosis of acute prostatitis, an antibiotic regimen was initiated. The selection of empirical antibiotic therapy considered the pharmacological interaction between levetiracetam and ciprofloxacin, and, therefore, second-line antibiotic therapy with sulfamethoxazole/trimethoprim was chosen [6,7]. The patient was discharged from the ER, and outpatient follow-up was scheduled.

**Figure 1:**
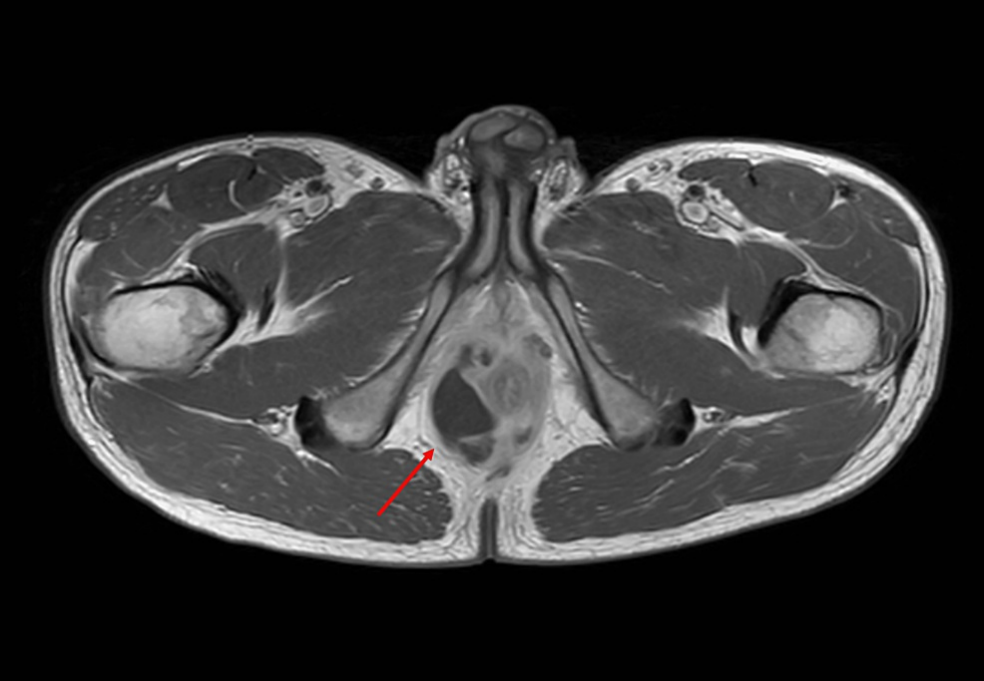
Abscess-forming prostatitis originating from the right lobe.

**Figure 2:**
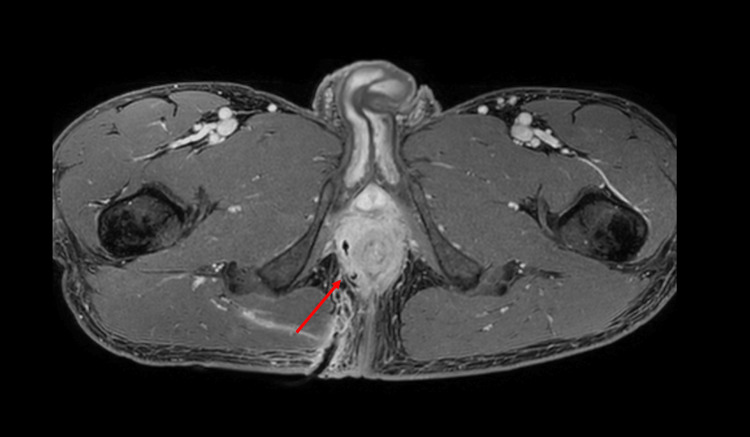
Abscess involution after drain insertion.

After self-readmission two days later due to exacerbation of anogenital pain, in-patient hospital care was required, and antibiotic therapy was escalated to ceftriaxone intravenously. Meanwhile, microbiological analyses showed bacterial growth of *Escherichia coli* (sensitive to ceftriaxone and sulfamethoxazole/trimethoprim, with no resistance to other antibiotics). Magnetic resonance imaging (MRI) was performed, which detected a complex abscess (35 × 21 × 36 mm) and fistulation system originating from the right prostatic lobe, reaching into the ischioanal fossa and circularly enclosing the anal canal (Figure 1). A prior transrectal ultrasound was deliberately not performed considering the recently operated hemorrhoids and the rapid availability of the MRI [8]. Based on this finding, a computer tomography-controlled insertion of a drain into the abscess was performed by the interventional radiologist. After consulting the in-house rheumatologist, adalimumab was paused. The antibiotic regimen was changed to sulfamethoxazole/trimethoprim via the oral route once clinical improvement, confirmation of *Escherichia coli* (still sensitive to ceftriaxone and sulfamethoxazole/trimethoprim) out of the abscess material, and a decrease of the inflammatory parameters in the blood examination had been observed. Additionally, a follow-up MRI five days after drain insertion confirmed a nearly complete regression of the abscess formation (Figure 2). Subsequently, the drain was removed the following day, and the patient was discharged.

After another six weeks of antibiotic therapy with sulfamethoxazole/trimethoprim, the patient reported no complaints in the anogenital region or signs of systemic infection. The urological follow-up after six weeks presented no sonographic (transabdominal) anomalies of the urinary tract. Thus, the antibiotic therapy was terminated after a total duration of eight weeks of antibiotic treatment.

## Discussion

We report the case of a 36-year-old male suffering from a bacterial prostatic abscess and a past history of hidradenitis suppurativa under treatment with adalimumab. This case report highlights a severe adverse effect of immunomodulating therapy and the importance of comprehensive anamnesis and clinical examination for accurate diagnosis.

TNF-α inhibitors have immensely improved the outcomes of inflammatory diseases such as hidradenitis suppurativa. Nevertheless, various side effects of blocking TNF-α and consequently reducing the expression of proinflammatory cytokines have been reported [4]. A large meta-analysis published in 2015 detected a significant risk of severe infection in patients suffering from rheumatoid arthritis associated with the use of biological therapies, including anti-TNF-α, anti-IL-1, anti-IL-6, and anti-CD20 [9]. On the contrary, a recently published systematic review and meta-analysis reported no significant risk of upper respiratory tract infections, nasopharyngitis, and influenza in patients treated with biologics for hidradenitis suppurativa [10].

Literature is scarce concerning the occurrence of bacterial prostatitis with abscesses associated with TNF-α inhibitors. El-Reshaid et al. reported a patient with a granulomatous, aseptic prostatic abscess during treatment with cyclosporin A. Under therapy with prednisone and mycophenolate mofetil, substantial involution of the abscess was accomplished [11]. Contrary to our case, it was a non-infectious abscess and a considerably longer disease course.

Even though there is no definitive proof of direct causality between the intake of adalimumab and the occurrence of abscess-forming prostatitis in this case, TNF-α most probably played a role in this case. The causality is supported by two other case reports of abscess-forming diseases under therapy with TNF-α inhibitors [12,13].

If continued therapy is required due to a severe disease course of hidradenitis suppurativa, medication without an immunosuppressive effect, for example, acitretin or dapsone, can be considered [14,15].

## Conclusions

This case presentation highlights a rare adverse event of the therapy with biologicals for inflammatory skin disease, leading to the development of complicated acute prostatitis in a young patient. This report may be useful in providing physicians with added insights into the management of similar complications in patients being treated with immunomodulating medications. This case report highlights the importance of critical evaluation and risk-benefit analysis before administering TNF-α inhibitors. Additionally, this study emphasizes the need for thorough examination and consideration of all differential diagnoses before administering surgical therapy for suspected symptomatic hemorrhoidal disease.
